# Editorial

**Published:** 1860-03

**Authors:** 


### Editorial.

### CRYSTAL GOLD.

We have recently been using Crystal Gold from the manufactory of A. J. Watts, which works better than any of his make we have before seen. It is but due to say for the past two years we have used but little crystal gold, having used the crystal gold foil, more than any thing else, or all other things together ; but for the past month we have been using the crystal gold ; the preparation we are using now is very tenacious and adhesive, and welds perfectly, when in a proper condition, and skillfully manipulated.

A. J. Watts is giving his entire attention to the production of crystal gold, and has brought it as near perfection as we can well conceive, yet we dare not say that much will not yet be accomplished. Ten years ago the man would have been thought insane who would have predicted the forthcoming of crystal gold. We have come to the conclusion to use the best of every thing in the practice of our profession that we can get, and expect something better in the future. T.

---

Elements of Somatology : A Treatise on the General Properties of Matter. By Geo. Macintosh MacLean, M. D.

Those who wish to obtain clear ideas of the general properties of matter should by all means read this little book. It constitutes a valuable introduction to a course on either Natural Philosophy or Chemistry. The ideas are happily arranged, and clearly stated. The author is evidently at home on the subjects treated. Our space is limited or we would enumerate the contents of the work at some length. We trust that all our readers will treat themselves to a copy of the work. W.

---

### EXCAVATORS AND PLUGGERS.

The character of the operations upon the natural teeth, particularly filling, depends much upon the instruments employed—excavators and pluggers.

That there has always been a want of perfection in these instruments, has been the unpleasant experience of all good operators. There have been deficiencies in many particulars : in the quality of material used, in the manner in which it is wrought, in the form of the instruments, a want of delicacy and finish, temper, &c. These objections are almost wholly overcome in some instruments we have been using for three months past, made by an eastern manufacturer, and recently introduced to the notice of the profession.

These instruments, both excavators and pluggers, excel any thing we have before seen. They seem to be made of the best material, and have a superior temper ; they retain an edge much longer than any insruments we have ever used, they have a delicacy and perfection of form hitherto unattained. Of the plugging instruments there seems to be quite a good variety ; but of the excavators there are very few forms ; it is true those he has made are the most important, but there are some very valuable forms he has not among his stock, at least that we have seen, but we are assured he will soon introduce every valuable variety. No one who uses them will for any consideration be without them. The price is about double that of the ordinary instruments of the same kind, yet they are cheaper. T.

### A NEW SOCIETY.

On the first Tuesday of January we had the pleasure of attending a meeting of the Mad River Valley Dental Association, at Xenia, Ohio. This is a society recently formed, but one which gives promise of much usefulness. It is composed of members of the profession from Bellefontaine, Liberty, Urbana, Springfield, Dayton and Xenia. These brethren have gone to work in earnest and with a determination to produce something valuable, and we have full confidence that it will be done. They all have a disposition to work ; and when that is the case, any society will be interesting and instructive. At the meeting which we attended, an interesting paper—which we hope to have for publication in the Register

—was read, on filling teeth; the subject was then taken up and discussed at some length, and several new ideas elicited.

After this a code of office ethics and fee bill was presented, both of which after due consideration were adopted. The code contains many excellent points, principles which should be adopted everywhere. The fee bill had an upward *tendency*, a fact which we are pleased to see, for when good prices are adopted, then time, energy and skill can be expended for the perfection of operations, and as a general thing, without it, will not be done.

Several new members were elected. All the members exhibited zeal; and we have no hesitation in saying, that much good will result from this society. It meets again in Urbana the first Thursday of April. T.

### AROMATIC TINCTURE OF MYRRH.

This is a fine preparation of Tincture of Myrrh aromatized. It is a good preparation for treatment of the teeth and gums; it is far more pleasant than the simple tincture; it is applicable in all cases where myrrh is required; it is to some extent deordorizing, and will in many cases destroy the offensiveness of bad breath. It is a good preparation for the dentists' use. It is prepared by Suire, Eckstein & Co., of this city, is put up in four ounce bottles, and neatly labeled. It may be used to moisten the ordinary dentrifice, just before using with the brush. Those who have used it will not be without it. T.

### SCIENTIFIC AMERICAN.

Hon. Judge Mason, of Iowa, who made himself so popular with the inventors of the country while he held the office of Commissioner of Patents has, we learn, associated himself with Mann & Co., at the Scientific American office, New York.

